# Immunoregulatory functions of immune complexes in vaccine and therapy

**DOI:** 10.15252/emmm.201606593

**Published:** 2016-08-29

**Authors:** Yu‐mei Wen, Libing Mu, Yan Shi

**Affiliations:** ^1^ Key Laboratory of Molecular Medical Virology, MOE/MOHSchool of Basic Medical SciencesShanghai Medical CollegeFudan UniversityShanghaiChina; ^2^ Center for Life SciencesDepartment of Basic Medical SciencesInstitute of Immunology Tsinghua UniversityBeijingChina; ^3^ Department of Microbiology, Immunology and Infectious DiseasesUniversity of CalgaryCalgaryCanada

**Keywords:** adjuvant, FcγR, immune complex, immune therapy, vaccine, Immunology

## Abstract

Clinical and experimental preparations of IgG/soluble antigen complexes, as well as those formed following antibody therapy *in vivo,* are multifaceted immune regulators. These immune complexes (ICs) have been tested in humans and animal models, mostly in forms of experimental or clinical vaccination, for at least a century. With intensified research on Fcγ receptor‐mediated immune modulation, as well as with immune complex‐directed antigen processing, presentation, and inflammatory responses, there are renewed interests of using ICs in vaccines and immunotherapies. Currently, IC‐based immune therapy has been broadly experimented in HBV and HIV viral infection control and antitumor treatments. However, mechanistic insights of IC‐based treatments are relatively recent subjects of study; strong efforts are needed to establish links to connect laboratory findings with clinical practices. This review covers the history, mechanisms, and *in vivo* outcomes of this safe and effective therapeutic tool, with a clear aim to bridge laboratory findings with evolving clinical applications.

**Glossary**
Self‐tolerance breaking: While immune tolerance refers to the lack of immune response to an antigen as a result of central (thymic selections) or peripheral (lack of co‐stimulation) tolerance education, an extrinsic trigger, such as the same antigen presented to the host in an immune complex, can initiate specific immune recognition against the tolerized antigen. The end results can be detrimental in cases of autoimmunity, or beneficial, as in the immune attack on tumor antigens.
Cross‐presentation: The ability of antigen contained in endo/phagocytic vesicles to be presented by MHC class I molecules on the APC surface. This is in contrast with the conventional understanding that class I antigens originate from endogenous protein synthesis. This is an intensely studied and contested topic, as several leading hypotheses, including models of “ER–phagosome fusion”, “endocytic/plasma membrane recycling”, and “cytosolic translocation”, are still being verified.
Toxoids: Richard Friedrich Johannes Pfeiffer classified bacterial toxins into endotoxins that were believed to be sequestered by the cell wall and exotoxins that were thought to be released into the surroundings. Those toxins, particularly the latter, can be thermally or chemically deactivated, that is, with formalin, to produce toxoids to be used as a form of deactivated vaccine. This is an old term with decreasing popularity in modern literature.
Fc and Fcγ receptor (FcγR): Fc, fragment crystallizable region, simply refers to the constant region of antibodies of any type. FcγRs are the cell surface receptors expressed mostly by cells of the immune system that have binding specificities to the Fc region of γ‐immunoglobulins.
Biological retention: Refers to any substance retained in a biological system. Used in the context of antibody induction or antibody‐based treatments, the term usually refers to the deposition of IC in tissues that can either serve as a source for persistent antigenic stimulation, usually with desirable outcomes, or induce uncontrolled immune attacks, as often seen in autoimmunity.
Plasmablast: Less mature plasma cell precursors, which could still undergo cell division.
Chemotaxis: Generically refers to the movement of a biological entity in response to chemical stimulus in their environment. Immunologically, this term refers to the immune cell movement driven by their surface expression of chemotactic receptors to or away from a gradient of cognate chemokines.
Erythroleukemia: A form of acute myeloid leukemia that involves the cells that give rise to the erythrocytes.
Antigenemia: Persistence of antigen in circulating blood. Antigenemia is used as an indication for severity of infection or as a measurement for efficacy of immune intervention.

## Introduction

Immune complex, as a form of autoimmune disorder and a tool in clinical therapy, has been an important topic of modern immunology. Binding of antigen by antibody, an immunologically simple event, changes properties of the original ligand, resulting in modulated antigen processing, presentation, receptor signaling, and inflammatory responses. On a larger scheme, antibody immune therapy has become one of the successfully implemented biomedical technologies in modern health care. Immunoglobulin‐based new drugs such as rituximab, ipilimumab, and alemtuzumab are among the highly effective in cancer treatments (Sondak *et al*, 2011; Scott *et al*, 2012). Intravenous immunoglobulin, IVIG, is also a choice treatment of autoimmunity, pediatric infections, and antibody deficiencies (Schwab & Nimmerjahn, 2013). In contrast, use of classically defined IC represents an old subcategory among the list of options. ICs in vaccine development and clinical interventions are becoming increasingly sophisticated, largely attributable to the newly gained understanding of Fcγ receptors (FcγR; Nimmerjahn & Ravetch, 2008; Ravetch, 2010). However, IC‐mediated functions are not solely controlled via those receptor engagements. For instance, its ability to transport antigen is also a consideration of anatomic location; its roles at different stages of immune induction and memory formation are modulated by additional spatiotemporal factors.

Over 100 years ago, IC vaccines were started as a practice of using antisera complexed to toxoids of *Corynebacteria diphtheria* or *Streptococcus pyogenes* to decrease the side effects in human vaccination (Copeman *et al*, 1922; Olitzki, 1935). One concern of this approach was that antigens coated with antibodies might have reduced exposure of surface sites, leading to limited antibody production. This concern was proven unnecessary by subsequent experiments (Olitzki, 1935). In 1950s, Terres *et al* reported that mice immunized with human or bovine serum albumin were sensitized faster if a specific rabbit antibody was also introduced to the recipients, an effect not seen with antigen alone or in combination with a control antiserum (Terres & Wolins, 1959a, 1961). The enhancement was optimal in a slight antigen excess (Terres & Wolins, 1959b, 1961; Terres *et al*, 1960). After another three decades, using peptides from HIV gp120 to rejuvenate CD4 T cells in HIV carriers, Berzofsky *et al* reported that addition of antibodies recognizing these peptides substantially increased the proliferative index of CD4^+^ T cells in the patients (Berzofsky *et al*, 1988).

In the meantime, immune complexes were casted in a negative shadow, mainly because of their frequent presence in sites of autoimmunity and inflammation, such as lupus and membranous nephropathy. Collectively called type III hypersensitivity, IC deposition at rates faster than their clearance leads to robust activation of the complement system and Fc receptor signaling. As consequences, mononuclear cell degranulation, antibody‐dependent cellular cytotoxicity (ADCC), and release of proinflammatory cytokines may result (Nydegger, 2007). Some efforts were made to characterize the pathogenic ICs. It was found that the IC‐mediated attacks were mainly driven by inflammatory cytokines such as TNF‐α (Warren *et al*, 1989). It was also found that ICs formed with low affinity antibodies tended to be present in high levels in the circulation and deposited more readily into subendothelia of renal capillary loops (Koyama *et al*, 1978). This was confirmed by mice genetically bred to produce only low and high‐affinity antibodies, as ICs chronically formed in the former were deposited in glomeruli (Devey & Steward, 1980).

The early work was not illuminated from mechanistic insights. A landmark turning point for IC's immune regulatory function was the discovery of FcγRs. In an attempt to understand the molecular nature of antibodies adsorbed to splenocytes reported by Boyden & Sorkin (1960), Belkin and Benacerraf suggested in 1966 that this affinity was mainly afforded by the heavy chain of antibodies, implicating a structural presence on the surface of macrophages interacting with the Fc portion of IgG (Berken & Benacerraf, 1966). In the 1980s, all common forms of FcγR were identified (Ravetch & Kinet, 1991), which subsequently developed into the current paradigm of Fc receptor biology (Nimmerjahn & Ravetch, 2007). Mouse activating FcγR family consists of FcγRI, RIII, and RIV, while human counterparts include FcγRI, IIa, IIc, IIIa, and IIIb. Except for human FcγRIIIb, these receptors transmit activating signals via an ITAM motif on the associated common γ‐chain or intrinsically present in their cytoplasmic domains. Both species have an additional inhibitory receptor, FcγRIIb, that signals via a cytoplasmic ITIM domain. Meticulous work by Ravetch *et al* gradually delineated the subtype specificities and binding strengths to IgG subtypes. In addition, the presence of lectin‐based type II FcγRs (DC‐SIGN and CD23) that are sensitive to the glycosylation state of Fc has been demonstrated in recent years (Ravetch, 2010; Bohm *et al*, 2014). Relevant to ICs, FcγR signaling has implications in at least three aspects: (i) the activating and inhibiting receptors, their affinities, and the emerging lectin‐based type II FcγRs; (ii) the subclasses of antibodies binding to these receptors; and (iii) the highly diverse expression of these receptors on host cells and their levels of expression at different stages of immune activation. These considerations are becoming indispensable to modern applications of ICs (Nimmerjahn & Ravetch, 2005, 2008; Guilliams *et al*, 2014). Under this backdrop, this review aims to provide a utilitarian look on ICs from a perspective of clinical application, to complement the current picture of antibody immune therapy.

## Mechanisms of immune regulatory functions of IC

### Cross‐presentation and cellular immunity

For extracellular antigens, the default antigen presentation pathway takes in soluble cargos via endocytosis, processed in progressively acidic endolysosomal vesicles by pH‐dependent chain activation of cathepsins (Amigorena & Savina, 2010), for loading onto the class II molecules. Solid particles engulfed via phagocytosis or soluble cargos endocytosed via receptor‐mediated uptake can be routed into MHC class I cross‐presentation via several mechanisms (Lizee *et al*, 2003; Burgdorf *et al*, 2007; Joffre *et al*, 2012). The latter activates CD8^+^ T cells, known as the cross‐presentation, that becomes the mechanistic basis of cellular immunity against antigens originated outside antigen‐presenting cells (APCs).

The involvement of ICs in cross‐presentation was initially noted in an assay to study the *in vivo* tumor killing by antibodies. EG7 tumor was inhibited or eradicated with an antibody against CD4 expressed by these cells. The antibody did not kill the tumor directly; the effect was entirely dependent on CD8^+^ T cells and FcγR γ‐chain (Vasovic *et al*, 1997). A logical explanation today is that binding of CD4 by the antibody facilitated tumor antigen processing by DCs and drove antigen‐specific CD8^+^ T‐cell activation. As a vivid confirmation, in the RIP‐mOVA mice whereby OVA is transcriptionally controlled by rat insulin promoter, infusion of OVA‐specific CD8^+^ T cells (OT‐I) was not efficient to trigger destruction of β‐cells. Addition of an OVA‐specific IgG antibody, however, led to severe autoimmune diabetes, suggesting that the presence of ICs can overcome the tolerance to “self” antigens. As expected, the breakdown of self‐tolerance required the presence of FcγR common γ‐chain (Harbers *et al*, 2007).

Cross‐presentation of external antigens is enhanced by antigen association with surface receptors on DCs (Shakushiro *et al*, 2004; Burgdorf *et al*, 2007; Chatterjee *et al*, 2012). Independent studies arrived at a similar conclusion that the presence of ICs facilitated the antigen uptake by two to three logs (Amigorena & Bonnerot, 1999; Schuurhuis *et al*, 2002). However, the real enhancement in antigen presentation measured by the degree of T‐cell activation was in the order of 4–5 logs (Regnault *et al*, 1999). γ‐chain ITAM domain‐mutant NOTAM mice have normal surface expression of FcγRs. This to some extent rescued the IC uptake; however, cross‐presentation of OVA IC was lost (Boross *et al*, 2014). That report seems to indicate that the ITAM is not required for uptake and yet behaves in part to direct intracellular antigen trafficking. Interestingly, in DCs, Syk deficiency downstream of ITAM phosphorylation disrupted not only cross‐presentation of IC antigen but antigen uptake as well (Sedlik *et al*, 2003). The difference is likely due to the fact that Syk is associated with a broader spectrum of inhibition on phagocyte signaling (Mocsai *et al*, 2010). Deep mechanistic understanding of how ICs facilitate cross‐presentation is currently limited. In one report, IC‐mediated enhanced cross‐presentation required TAP proteins and was sensitive to proteasome inhibition, suggesting the involvement of cytosolic targeting of internalized antigens (Regnault *et al*, 1999). In some systems, IC‐mediated antigen uptake retained the complex at early endosome, rather than permitting further maturation to more degradative lysosome (Chatterjee *et al*, 2012; Cohn *et al*, 2013). Although the molecular nature of this “shallow” targeting is unknown, the findings are in agreement with the contemporary understanding that reduced cargo digestion is the main advantage of DCs in antigen presentation over other APCs (Joffre *et al*, 2012; Kotsias *et al*, 2013; Hari *et al*, 2015).

CD8^+^ DC subsets in the mouse periphery are usually considered to be superior in cross‐presentation, and a developmentally overlapping population of CD103^+^ DCs is known for similar functions. These cells are phenotypically mirrored by human CD141^+^ DCs (Platzer *et al*, 2014). However, how these cells are involved in IC‐mediated cross‐presentation is not well understood (Joffre *et al*, 2012). Not in strict sense an standard IC, OVA linked to an antibody specific for a C‐type lectin receptor DNGR‐1 found on CD8^+^ conventional DCs elicited strong CD8^+^ T‐cell activation and eliminated OVA‐positive B16 tumor *in vivo* (Sancho *et al*, 2008). Yet, when delivered into the host, OVA conjugated to anti‐DCIR‐1 and anti‐DEC205, expressed by CD8^−^ and CD8^+^ DCs respectively, suppressed OVA‐expressing B16 tumor to a similar extent (Neubert *et al*, 2014). As IgG1 antibodies used in this study carried Fc mutation (N297A) that disrupts FcγR binding, both preparations might function merely as a delivery vehicle. This paper therefore did not address the effect of FcγR signaling in cross‐presentation. In another report, Bevan's group suggested that FcγR‐mediated IC cross‐presentation was only essential in CD8^−^ DCs, while IC uptake and processing by CD8^+^ DCs were not affected by the absence of FcγRs (den Haan & Bevan, 2002).

DCs can rapidly engulf ICs and sustain the stimulation capacity for a long period after a short pulse (Bonifaz *et al*, 2004). In influenza virus‐challenged mice, the absence of extracellular availability of ICs did not strongly affect the primary CTL response to flu NP antigen. However, the recall response was limited. Transfer of immune serum from control‐infected mice restored the long‐term CD8^+^ T activation to NP antigen (Leon *et al*, 2014). Crossing μMT (B‐cell‐deficient) mice to γ‐chain knockout mice, NP‐specific CD8 response upon viral challenge was reduced. Transfer of immune serum restored both primary and secondary responses in μMT mice, yet failed to do so in the double‐deficient recipients, suggesting the essential role of FcγR signaling in cross‐presentation. This observation was confirmed by a recent study that the ICs associated antigens were present in the endocytic vesicles from early endosome to lysosome one day after the uptake, with dissociation of antigen taking place along the route (Liu *et al*, 2016).

### Helper T cells and antibody responses

FcγR‐mediated IC signaling likely supports a simultaneous activation of both CD8^+^ and CD4^+^ T cells. For instance, prostate‐specific antigen‐containing ICs, when targeted via FcγR, led to a combined activation of both CD4^+^ and CD8^+^ T cells, while mannose receptor targeting was more associated with CD4^+^ T‐cell response alone (Berlyn *et al*, 2001). Regarding intracellular antigen trafficking, Syk signaling is an essential event for efficient MHC class II epitope peptide loading, as in the case of cross‐presentation. A mutation in FcγR that disrupts Syk association failed to deliver internalized antigen in ICs to lysosome (Bonnerot *et al*, 1998). Syk deficiency resulted in reduced MHC class II/DM molecule association with internalized antigens (Le Roux *et al*, 2007). As evidence of IC‐facilitated class II antigen presentation, a viral glycoprotein rabies G as a free antigen entered Rab5^+^ early endosome with a kinetics similar to that of antibody (ARG1)‐bound protein. Thereafter, the IC became localized to Rab9^+^ late endo/lysosome but at no time was co‐localized with Rab11 (slow recycling endosome) and Rab4 (rapid recycling endosome) markers, while the free antigen was more associated with Rab11^+^ vesicles (St Pierre *et al*, 2011). Therefore, ICs may facilitate antigen delivery to vesicles in late endocytic maturation steps for MHC class II presentation. This notion inevitably disagrees with the “shallow” targeting reported in cross‐presentation as discussed previously. This discrepancy possibly resulted from different antibodies used in experiments, which could have preferences in FcγR subtype association, leading to distinct routes of trafficking. However, considering ICs in general enhance both class I and class II antigen presentation, one can postulate that binding of antibodies in a uncharacterized manner intercepts the robust proteolytic “grinding” in the endolysosomal system, saving antigens at multiple stages for eventual surface presentation.

One main mechanism for enhanced antigen presentation of ICs to B cells is longer biological retention. This is most evident in the trapping of ICs by follicular dendritic cells (FDCs) (Szakal *et al*, 1988a,b; Kosco *et al*, 1989). It was reported that great quantities of ICs attached to the long membrane processes of FDCs (Szakal *et al*, 1988b). FDCs have high FcγRIIb expression at protein level (Qin *et al*, 2000) and reports suggested that ICs were internalized by FcγRIIb (Bergtold *et al*, 2005), consistent with the observation that FDCs and B cells only express this Fc receptor. The ICs internalized by FcγRIIb were retained in the cytoplasm and antigens within the complex were kept in their native form and were made available to B cells via surface recycling (Bergtold *et al*, 2005). The exact order of baton exchange has been reported. ICs were initially trapped by lymph node subcapsular macrophages; B cells then acted as an intermediator fetching and sending the complexes to FDCs (Phan *et al*, 2007). FDCs received complement‐coated ICs from non‐specific B cells, and following endocytosis sorted, the complexes to a non‐degradative compartment for extended resurfacing (Heesters *et al*, 2013). Furthermore, FDC‐associated ICs played a role for BCR hypermutagenesis to generate high‐affinity antibodies (Wu *et al*, 2008).

Type II FcγRs are also implicated in B‐cell activation. IgG is glycosylated at asparagine 297 with a biantennary core glycan that serves as the base for further addition of sugar moieties, including fucose, galactose, bisecting *N*‐acetylglucosamine, or sialic acid, etc. This complex glycosylation event controls IgG conformational state and determines the preferential binding to either type I or type II FcγRs. For instance, sialylation reduces the binding of Fc to all type I while increasing the binding to type II receptors (Pincetic *et al*, 2014). Treatment to remove this moiety suppressed ankle swelling in a model of serum transfer arthritis and prolonged survival of a mouse strain from an autoantibody‐induced lupus‐like disease (Albert *et al*, 2008). Targeting OVA to DCs via DEC‐205 without concomitant activation via CD40 induced IgG1 antibody response and but a suppressed DTH (delayed type hypersensitivity) response. When the antiserum from those mice was transferred to new hosts that were challenged with the antigen in the full complement of anti‐CD40, an extensive reduction of antigen‐specific IgG1, IgG2b, and IgG2c was detected, accompanied by a reduced DTH response as well. These results suggested that antibodies produced in the original hosts without DC activation signal were immunosuppressive. Indeed, the antibody produced without anti‐CD40 had a N297 sialylation rate higher than those with the treatment, due to higher levels of sialyltransferase in the plasma cells (Oefner *et al*, 2012). As this reduction in antibody production and DTH response was independent of FcγRIIb, the involvement of type II receptors was implicated (Oefner *et al*, 2012). Type II FcγR engagement is an important consideration in vaccination. In a trivalent inactivated influenza vaccine (TIV)‐immunized individuals, mass‐spec analysis did not show a significant glycosylation pattern change in the recipients. Interestingly, the subtle differences in glycosylation in the existing antibody pool were found to be a predictor of responders and non‐responders to the vaccine (Wang *et al*, 2015a). In addition, it was recently reported that in human volunteers, TIV led to a peak production of HA‐specific sialylated IgG from plasmablast one week after the immunization, followed by a drop in abundance by week three. This elevation was resulted from glycosyltransferase expression in these early responding B cells. Importantly, this sialylated IgG, via CD23, triggered FcγRIIb expression, which is a known threshold setter for BCR signaling (Wang *et al*, 2015b) resulting in altered antibody‐binding affinity. The pattern of Fc glycosylation is associated with protection efficacy of a given antibody. Palivizumab, a humanized mAb against respiratory syncytial virus (RSV) derived from animal cell culture, has a heterogeneous glycosylation pattern. The same antibody derived from plant cells (*Nicotiana benthamiana*) that had their xylosyl‐ and fucosyltransferase activities deleted produced G0‐dominated glycans, palivizumab‐N. This form of glycosylation showed enhanced FcγRIII binding and better protection against RSV challenge (Hiatt *et al*, 2014).

### Regulation of antigen‐presenting cells

ICs can directly activate APCs via activating FcγRs. Upon IC binding to these receptors, the ITAM/Syk interaction is the primary signaling axis in phagocyte activation, leading to the full spectrum of signaling events involving PI3Ks, PLCs, MAPKs, and NF‐κB (Mocsai *et al*, 2010). Accordingly, ICs can trigger DC expression of CD40, CD86, IL‐6, IL‐13, and unexpectedly IL‐2 (Matsubara *et al*, 2006; Perreau *et al*, 2008; Boross *et al*, 2014). In addition, ICs themselves were sufficient to trigger CD11c^+^ DC aggregation and entrance into lymphatic vessels for migration to LNs in a CCR7/MMP‐9‐dependent manner (Clatworthy *et al*, 2014). While in general the activation of APCs is linked to the FcγR signaling, it has been recently suggested that once the ICs are inside the cell, cytosolic antibody receptor TRIM21 can bind to the Fc portion and activate a K63‐specific Ubc13‐mediated ubiquitination event. This event can independently activate NFkb, AP‐1, and IRF pathways, adding another level of complexity to IC signaling (McEwan *et al*, 2013).

Immune‐suppressive effects of ICs are equally noted. The small immune complexes formed in IVIG can inhibit macrophage responses to IFN‐γ, an effect mainly mediated by IgG Fc binding to FcγRIII, and surprisingly independent of FcγRIIb (Park‐Min *et al*, 2007). In complement C5a‐induced inflammatory recruitment, IgG1 ICs suppressed the neutrophil and macrophage chemotaxis. This inhibition was a result of an unexpected collaboration between ITIM tyrosine phosphorylation in FcγRIIb itself and a ITAM phosphorylation of Syk downstream of dectin‐1 (Karsten *et al*, 2012). In contrast to the conventional understanding that ICs formed in chronic infections cause inflammatory response by FcγR cross‐linking, it was recently found that in mice with clone 13 LCMV (long term) infection, the presence of ICs resisted CD4^+^ T‐ and B‐cell depletion by anti‐CD4 and anti‐CD20 (rituximab) antibodies and blocked the activation of DCs by agonistic anti‐CD40. The effect can be explained by competitive inhibition in the long‐term presence of excessive ICs (Wieland *et al*, 2015). A similar report in the same issue of immunity confirmed the finding and demonstrated additional defects in cross‐presentation. It was shown in the latter that the suppression was dependent on CD4^+^ T‐supported antibody production (Yamada *et al*, 2015). This conclusion somewhat echoes the observation that in influenza infection, peak of antibody production formed ICs that blocked antibody‐dependent phagocytosis (Astry & Jakab, 1984), both pointing to IC's negative impact on immune activation.

**Figure 1:**
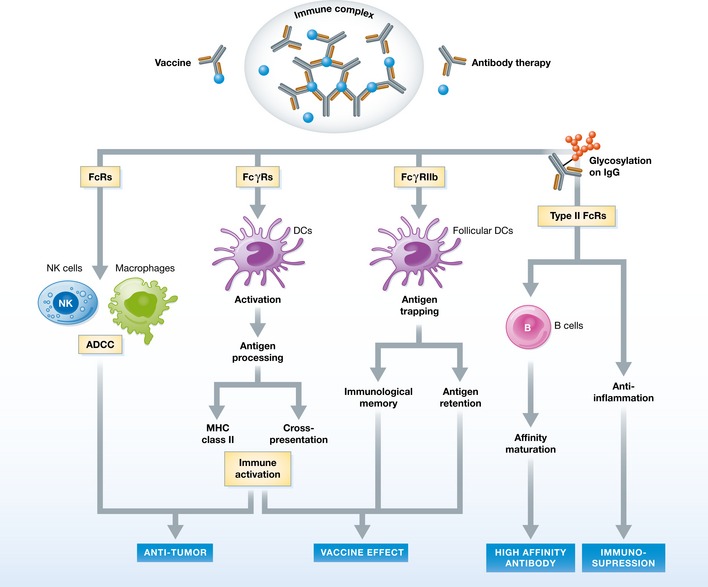
Immunologic regulation of ICs in vaccine and tumor therapy ICs preformed as vaccine preparations or as a consequence of antibody binding to endogenous antigens can signal via activating receptors to induce ADCC in macrophages and NK cells, leading to lysis of tumor or infected cells. The same signal via FcγRs on dendritic cells results in enhanced antigen uptake and upregulated antigen presentation, both to MHC class II‐restricted CD4^+^ T cells and to CD8^+^ T cells via cross‐presentation. ICs retained in lymphoid tissues via deposition or FcγRIIb‐mediated endocytosis extend the antigen availability for B‐cell activation, resulting in increased antibody response and immune memory induction. Different glycosylation patterns on Fc regulate the preferential binding to type I or type II FcγRs, controlling the state of inflammation. Sialylation of Fc with preferential binding to type II FcγR in particular helps to set the threshold for the production of high‐affinity antibodies.

**Figure 2:**
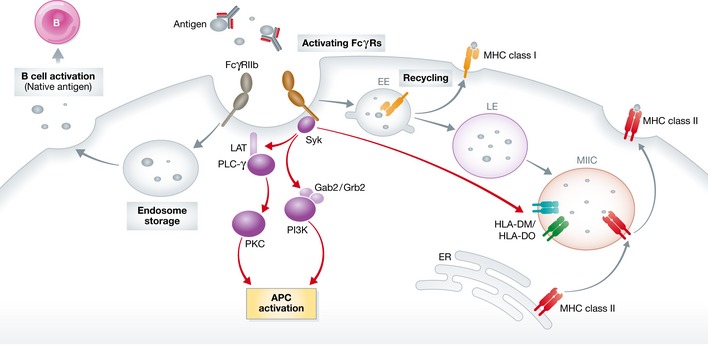
FcγR ligation and the fate of antigens Antigens in ICs entering APCs via different FcγRs show distinct trafficking patterns. In general, FcγRIIb‐mediated uptake traps the cargo in non‐degrading vesicles for prolonged extracellular release of antigens in their native forms. This feature is important for B‐cell activation. Activating FcγR‐mediated entry may direct the cargo into two routes. One is through the shallow early endosomes whereby the antigens are recycled to the cell surface in complex with MHC class I molecule for cross‐presentation. The other route leads the antigens to the MHC class II compartment (a branch of late endo/lysosomal compartment) and epitope peptides generated in this harsher environment are loaded onto MHC class II molecules for conventional CD4 T‐cell activation. The ITAM motif present in FcγRs (intrinsic or in the common γ‐chain) recruits Syk and activates signal transducers of PLC, PKC, and PI3K leading to APC activation. Syk signaling may assist the class II peptide exchange regulated by class II‐like DM/DO molecules in the MHC class II compartment.

To summarize the discussion on the immune regulatory functions of IC engagement of FcγRs, Fig 1 categorizes the immunologic effector functions of IC uptake by DCs and macrophages, and Fig 2 illustrates the known APC functional regulations and antigen processing pathways.

## Experimental and clinical applications of ICs

### IC vaccines in persistent viral infections

In HIV infection, experimental work on ICs started by using antibodies targeting the viral epitopes, as an approach to form ICs *in vivo*. ICs formed by antibodies recognizing HIV CD4 binding sites (CD4bs) and V3 region of gp120 have been promising, eliciting strong neutralizing antibodies against the virus in mice (Visciano *et al*, 2008; Hioe *et al*, 2009). One of V3 region‐specific antibodies mAb 654‐D showed high affinity to large variants of gp120 subtypes. The binding of this antibody significantly increased the accessibility of V3 region by other mAbs, suggesting that the attachment of 645‐D opened up the V3 region that is masked by other structures, a common detection‐averting strategy of HIV (Kwong *et al*, 2002; Pan *et al*, 2015). This IC elicited anti‐V3 neutralizing antibody in BALB/c mice (Hioe *et al*, 2009). IC formed with mAb A32, binding to gp120 outside the CD4 binding site, induced neutralizing antibodies against a panel of HIV strains, although the enhancement over gp120 alone was not pronounced. In the presence of cholera toxin and LPS as adjuvants, the breadth of the resulting antisera in neutralizing diverse HIV isolates was widened (Liao *et al*, 2004).

Recently, broadly neutralizing antibodies (bNAb) against HIV‐1 have seen great success. bNAb‐producing B cells identified in HIV carriers by high‐throughput screening are capable of neutralizing a large number of HIV isolates (Walker *et al*, 2009; Wu *et al*, 2010). To eradicate the latent pool of HIV in the carriers, one strategy was to reactivate the latent virus in the presence of antiretroviral treatment followed by immune therapies. bNAbs were shown to suppress HIV viremia in humanized mice and reduced the HIV DNA in the infected lymphocytes (Horwitz *et al*, 2013). However, the generic treatment was followed by viremia rebound after termination, suggesting the presence of a persistent pool of cells with intact virus. In humanized mice, delivery of bNAb cocktail (TriMix antibodies with 3BNC117, anti‐CD4bs; PG16, anti‐V1/V2 loop region; and 10‐1074, anti‐V3) 4 days after HIV inoculation significantly delayed the viremia rebound, with a statistically significant increase in aviremic period. Furthermore, TriMix bNAbs with a combination of three viral inducers (shock and kill) significantly suppressed viremia rebound over a long period of up to 105 days in comparison with antibody alone or with in combination with a single inducer (Halper‐Stromberg *et al*, 2014). Implicating the role of ICs, TriMix antibodies carrying Fc mutation that disrupts human and mouse FcγR binding showed shortened delay in viremia rebound (Halper‐Stromberg *et al*, 2014). The definitive proof of FcγR engagement by bNAbs was elegantly demonstrated in a recent study. Human antibodies with Fc domains swapped with mouse Fc subtypes were used in a model of HIV entry inhibition assay. While the Fc swapping minimally affected the Fab binding, mouse IgG2a Fc was found to be most potent in blocking HIV entry in an *in vivo* analysis. Accordingly, in mice lacking all FcR α‐chain or common γ‐chain/FcγRIIb, the advantage of IgG2a over a non‐FcR‐binding mutant Fc was lost (Bournazos *et al*, 2014). To mimic the human infection, a mouse model with all FcγRs replaced with human FcγRs was used. Human HIV‐specific IgG1 antibodies with Fc domain engineered to carry high‐affinity mutations to activating FcγRs (FcγRIIIa and FcγRIIa) were highly protective against HIV entry in comparison with Fc mutants that diminish the FcγR binding (Bournazos *et al*, 2014). Beyond direct eradication of HIV, in a mouse model to approximate human mother to child transmission, FrCasE virus‐infected neonatal mice develop erythroleukemia once reaching adulthood. Neonatal delivery of a specific mAb 667 boosted antibody protection and T‐cell response that resulted in survival of treated mice in comparison with the total mortality in untreated controls. This protection was mediated by ADCC and formation of virus/antibody ICs (Michaud *et al*, 2010).

Viral hepatitis B is another persistent infection treated with ICs as therapeutic vaccine. In a HBV vaccination model, ducklings were infected with duck hepatitis B virus (DHBV). They were treated with IC (DHBsAg complexed to rabbit anti‐DHBs) linked to *Staphylococcus aureas* bacteria as a solid matrix complex. About 70% and 50% of treated ducks were cleared of viremia and antigenemia after three injections (Wen *et al*, 1994). The efficacy of IC‐based vaccines in HBV infections was studied in mice in 1980s. ICs formed with non‐specific IgG or IgM antibodies showed minimal enhancement, as did the Fab fraction of those specific antibodies (Celis & Chang, 1984). HBsAg ICs stimulated T cell's avidity, which in turn induced antibody production *in vivo* (Celis *et al*, 1987). IC‐mediated effects were followed by a seroconversion to HBsAb positivity in HBsAg‐transgenic mice, indicating self‐antigen tolerance breaking (Zheng *et al*, 2001). Subsequently, DCs induced from chronic hepatitis B patient PBMCs with GM‐CSF/IL4 were stimulated by either HBsAg, antibody, or IC *in vitro*. Expression of functional markers including MHC class II, CD80/CD86, and CD40 was found increased only with the IC stimulation. Furthermore, when T cells from the patients were incubated with the stimulated DCs, higher levels of IFN‐γ and IL‐2 were observed in the IC‐stimulated group (Yao *et al*, 2007). Using a lentivirus vector coding HBsAg either alone or fused to Fc domain of mouse IgG2a, the fusion version induced much higher CD8^+^ T‐cell activation in recipient mice, as well as elevated CD4^+^ T‐cell response and antibody production (Hong *et al*, 2011). To simplify the vaccination protocol, Meng *et al* produced HBsAg‐Fc (from IgG1) fusion protein and used it to immunize HBsAg‐transgenic mice. This resulted in a predominant production of Th1 cytokines and reduced serum HBsAg level (Meng *et al*, 2012). Encouraged by these experimental findings, human HBsAg IC was adsorbed to alum and used to immunize 14 HBV chronic hepatitis B patients in a pilot study. The recipients showed substantial reduction in serum HBV DNA and HBeAg was converted to negative (Wen *et al*, 1995). In a phase 1 clinical trial, healthy subjects receiving various amounts of HBsAg IC preparations developed IgG1 and IgG3 antibodies against the immunogen with a simultaneous increase in serum IFN‐γ and IL‐2 (Xu *et al*, 2005). From this outcome, one of the doses was used to immunize chronic hepatitis B patients. Five of ten patients responded and showed ≥ 2 logs decrease in serum HBV DNA, with a loss or marked reduction of HBeAg and an appearance of anti‐HBeAg; and two of these patients developed anti‐HBsAg antibodies (Xu *et al*, 2005). In a phase 2 clinical trial, HBeAg‐positive chronic hepatitis B patients were immunized with six injections of the IC. At the end of post‐trial follow‐up, HBeAg seroconversion rate was 21.8% (17/78) and 9% (7/78) in the IC and placebo groups, respectively (*P* < 0.05; Xu *et al*, 2008).

### IC in other microbial infections

In addition to the vaccine trials against HIV and HBV, smaller scale efforts to control other infections have been reported. CTL response against influenza virus infection is reduced in aging populations. While young BALB/c mounted strong cytolytic response with no discernible differences in intensity to flu NP antigen following inoculation of live, killed H1N1 virus, or a preparation of the virus plus a NP‐specific antibody, only the IC significantly restored the same response in the aged mice. This enhanced killing was associated with increased IFN‐γ production in both CD4^+^ and CD8^+^ T cells (Zheng *et al*, 2007). Due to its rapid spread and high rate of mutagenesis, the emphasis of influenza infection control has been to generate bNAbs against conserved regions of hemagglutinin (HA) (Okuno *et al*, 1993, 1994; Ekiert *et al*, 2011). One version of the antibody, CT149, targeting the stem region of HAs of both type I and type II isolates blocked the membrane fusion. In addition, the binding by this antibody induced FcγR‐dependent ADCC, suggesting the involvement of ICs (Wu *et al*, 2015). For Ebola infection, several B‐cell clones of highly potent GP‐specific neutralizing antibodies from a disease survivor have been tested. These antibodies also mediated ADCC via FcγR as mutants in the Fc that interfered with the receptor binding lost the cytotoxicity (Corti *et al*, 2016). *Francisella tularensis* (FT) is the causative pathogen of tularemia. DCs coated with monoclonal antibody‐treated FT delivered intranasally protected hosts from subsequent challenges. This effect was not affected by the absence of FcγRIIb, suggesting a preferential engagement of activating FcγRs (Pham *et al*, 2014). In malaria infection, it was found that the FcγR binding was critical for the uptake of IC (McClintock *et al*, 2005). In one study, the mortality of *Plasmodium berghei* infection that is often lethal was prevented if the antiserum from immune mice was transferred into infected mice. This protection was eliminated in the absence of FcγR γ‐chain (Pleass, 2009). In addition, Phoolcharoen *et al* transiently expressed Ebola virus GP1 glycoprotein fused to an antibody heavy chain from a humanized GP1‐specific antibody in *Nicotiana benthamiana* (tobacco plant). The purified fusion protein induced high titers of Ebola‐specific IgG antibodies, similar to those induced by GP1 virus‐like particles, suggesting a scheme of vastly increased production capacity for IC preparations (Phoolcharoen *et al*, 2011). Urgent productions of ICs to protect populations under threat from rapidly emerging viral infections such as Middle East respiratory syndrome (MERS) are also being tested (Lu *et al*, 2015).

Although ICs are in general considered to be conducive to antiviral immunity, it should be noted that in some cases the presence of virus‐specific antibodies can increase infectivity, a phenomenon called antibody‐dependent enhancement (ADE) of viral infection (Tirado & Yoon, 2003). In the STEP trials, HIV *gag*,* pol*, and *nef* gene cassettes were inserted into the adeno vector, however individuals with preexisting immunity against the virus showed greater risk of HIV infection following the immunization (Buchbinder *et al*, 2008). The same adverse effect was seen for additional viruses, such as dengue (Goncalvez *et al*, 2007), measles (Iankov *et al*, 2006), and over a dozen others (Suhrbier & La Linn, 2003). FcγR‐facilitated virus entry and a skewed cytokine profile may be the main culprits. These are important considerations in assessing ICs' immune regulatory functions.

### IC in tumor immunity

Cancer immunotherapy has seen tremendous strides with technological developments in the immune checkpoint blockage. The basic concept is to reduce negative regulators of adaptive immunity, mainly targeting CTLA‐4 and PD‐1/PD‐L1. Another modality of antibody‐based cancer therapy is to target surface factors critical to tumor cell growth, such as EGFR and VEGF, of which optimized FcγR ligation is an important consideration (Nimmerjahn & Ravetch, 2012). Concurrent to these progresses, IC‐based intervention has remained an alternative due to its low side effects. Antibody binding to tumor surface antigens triggers ADCC and FcγR engagement is an important activation signal for tumor lysis (Moalli *et al*, 2010). A large number of experiments have been carried out using B16 or its variants as the tumor model. Here, ICs have been shown to mediate tumor suppression via prophylactic, therapeutic and memory‐inducing effects (Rafiq *et al*, 2002). In mice challenged with B16 tumor‐expressing OVA antigen (MO4), injection of DCs pulsed with OVA‐containing IC, but not with OVA or antibody alone, inhibited the tumor growth. Furthermore, treatment with DCs loaded with ICs cured 40% of mice bearing established tumors. Protection mediated by DC/IC immunization was long‐standing and induced memory responses. In recipients that had been vaccinated with OVA IC‐pulsed DCs, MO4 inoculation failed to establish the tumor (Rafiq *et al*, 2002). In addition, specific antibody targeting HER2/neu antigen expressed by a whole‐cell vaccine (3T3‐neu/GM) was shown to enhance antigen‐specific CD8^+^ T‐cell activation and memory development and protected the hosts from the tumor challenge (Wolpoe *et al*, 2003; Kim *et al*, 2008)). Loading antibody against myeloma cell surface syndecan‐1, a heparan sulfate proteoglycan expressed on cancer cells, induced HLA A2.1‐mediated antigen presentation to MHC allele‐matched donors, resulting in cytotoxic T‐cell activation (Dhodapkar *et al*, 2002). Here, antisyndecan‐1 antibody served as a facilitator of overall antigen presentation by DCs. The ensuing cytotoxicity elicited from human HLA‐A2.1‐restricted CTLs was directed toward other tumor antigens, such as MAGE‐3 and NY‐Eso‐1(Dhodapkar *et al*, 2002).

FcγRs play an important role in IC‐mediated tumor immunity. In the syndecan‐1 experiment, it was found the antibody coating did not alter the level of uptake of tumor cells yet blocking of FcγRs significantly reduced antigen‐specific T‐cell proliferation (Dhodapkar *et al*, 2002), suggesting that the regulatory role of FcγRs is mostly at steps beyond opsonization, primarily at DC maturation. Preferential induction of tumor immunity can be achieved by fine‐tuning the signaling intensity ratios of activating vs inhibitory FcγRs. This concept was proposed by Ravetch *et al* (Nimmerjahn & Ravetch, 2005). For instance, a tumor‐specific antibody (TA99) was found to have mIgG2a Fc that binds to the activating FcγRs with much higher intensity than the inhibitory FcγRIIb. This preferential binding, particularly to FcγRIV, endowed a strong antitumor effect (Nimmerjahn & Ravetch, 2005). The signaling of FcγRs in IC‐mediated immune therapy can lead to a vaccinal effect, especially under properly selected FcγR engagement. In a recent paper, mice challenged with CD20‐positive EL4 tumor survived in the presence of mIgG2a CD20‐specific antibody. This protection was lost in the absence of total or all activating FcγRs. As expected, those primed mice developed an immune response that rejected a subsequent challenge of neo‐antigen CD20‐positive EL4 tumor (DiLillo & Ravetch, 2015). When the lymphocytes from primed mice or from mice with a DC‐specific deletion of FcγRIV were transferred into new hosts, the former transfusion showed a better tumor resistance than the latter in the naïve mice, suggesting the role of this receptor in mediating the vaccine effect (DiLillo & Ravetch, 2015). When the concept was tested in a humanized mouse system with the Fc region of mouse CD20‐specific mAb replaced with the human counterpart and a total replacement of mFcγRs with hFcγRs in the host, it was found that mutations in the Fc that allowed better binding to hFcγRIIIa and IIa, as compared to IIIa alone protected the host to the best extent (DiLillo & Ravetch, 2015). Overall, as in the case of antipersistent viral infections, the selective pairing of IgG FcγR subtypes *in vivo* may represent the most promising direction in IC‐mediated cancer therapy. Lastly, intracellular tumor antigens that are often shielded from immune recognition can become stimulatory in the presence of ICs. NY‐seo‐1 is a tumor antigen considered to be strictly intracellular. However, in combination with 5‐fluorouracil, which likely stresses tumor cells for ensuing releases of intracellular contents, injection of mAb against NY‐seo‐1 resulted in significant tumor growth inhibition. This specific combination caused antigen epitope spreading, whereby other intracellular tumor antigens also induced T‐cell activation (Noguchi *et al*, 2012).

## Future prospective

The demand to employ IC technology in disease prevention and treatment makes it an urgent task to study their effects in both basic and clinical settings (Table 1). As one of the oldest technologies of immune therapy, the track records of safety and simplicity of ICs are valuable on this day of enormous breakthroughs at other directions. The downside of IC‐based interventions is also clear: relative low clinical efficacy and often unpredictable clinical and experimental results. However, with growing understanding of FcγRs and their preferential bindings to IgG subtypes, and modern biomedical engineering to steer the desired pairing, the future outcomes will likely change. In hindsight, if these understandings were built into vaccine designs at the time, several large‐scale clinical trials might have seen different and likely more favorable effects. In the case of HIV prophylaxis, IC formation that results in the undesired cytokine profile and the existing antibodies targeting the adeno vector are two factors likely addressable via precision targeting of activating FcγRs. In IC‐based anti‐HBV vaccination and immune therapy, first‐hand monitoring of responder cells and their FcγR expression should be built into the protocols of next‐stage clinical trials. At this time of personalized medicine, IC preparations tailored to individual FcγR polymorphism could be within reach as well. Addressing FcγR binding is only a mandatory starting point. Additional efforts will be needed to understand the precise antigen trafficking as a consequence of antibody binding, as well as to map inflammatory pathways and tissue targeting. As the desired immune protections vary from pathogen to pathogen, and entry portals are also vastly different, specific IC preparations can be made to induce CD4/CD8 and antibody responses for optimal protection. For instance, for antitumor and antiviral immunity that relies on cytolytic T‐cell activation, an IC vaccine designed to target the “shallow” endocytic compartment will be highly ideal. Admittedly, IC‐mediated immune regulations have not been as intensely investigated as other classes of immune adjuvants. However, mechanistic research will be most crucial for future breakthroughs in IC‐based human disease intervention and is the strongest assurance to maintain the continuing development of this old biomedical technology.

**Table 1:** A select list of published laboratory and clinical assessments of IC‐based immune therapy and vaccination

Complex	Species	Disease targeted/model	Observations	References (authors, year)
Antitumor				
Specific mIgG2A/hCD20	Mouse (humanized)	EL4 expression hCD20	Tumor clearance; via ADCC; FcγRIV is required; engineered selective engagement of hFcγRIII is effective	DiLillo and Ravetch (2015)
Specific mIgG1/NYU‐seo‐1	Mouse	Colon epithelial tumor	Tumor inhibition; CD8 required; enhanced by chemotherapy; epitope spreading	Noguchi *et al* (2012)
Fusion of OVA to anti‐DEC205 antibody, DEC205 targeting etc.	Mouse	B16, HER2‐neu bearing tumor	Enhanced tumor resistance, involvement of both CD4 and CD8 responses, enhanced by anti‐CD40	Wang *et al* (2012); Charalambous *et al* (2006); Bonifaz *et al* (2004)
OVA epitope conjugated to anti‐DNGR‐1 (mouse or rat)	Mouse	B16	Tumor inhibition and prevention; metastasis inhibition; enhanced antigen uptake by CD8α^+^ DCs	Sancho *et al* (2008)
Specific mIgG2a mAb/HER2‐neu	Mouse	Her2 transgenic mouse	IC‐mediated uptake of antigen by DCs; specific CD8 expansion; Fc required	Kim *et al* (2008); Wolpoe *et al* (2003)
Polyclonal rIgG/cell surface OVA	Mouse	Self‐antigen tolerance breaking	Breaking CD8 tolerance of OVA transgene; requires FcγR γ‐chain and complement C3	Harbers *et al* (2007)
IgG2A (TA99)/Gp75	Mouse	B16	Tumor clearance; FcγRIV is required	Nimmerjahn and Ravetch (2005)
Specific mAb hIgG1 (B4‐B)/syndecan‐1	Human	Myeloma	CTL induction to unrelated testis antigen epitopes; reduced with anti‐FcγR antibodies	Dhodapkar *et al* (2002)
Specific rIgG/OVA	Mouse	B16 (OVA expressing)	Mouse survival; FcR‐γ chain is required, absence of FcγIIB reduces tumor burden	Kalergis and Ravetch (2002)
Specific rIgG/OVA	Mouse	B16 (OVA expressing)	Reduced tumor establishment; FcγR‐γ required; TAP and β2m required; MHC class II required	Rafiq *et al* (2002)
Vaccine				
bNAb/Ebola GP	Macaque	Ebola	Protection against Ebola challenge, ADCC	Corti *et al* (2016)
bNAb/flu HA	Mouse	Influenza model	Protection against influenza challenge, ADCC	Wu *et al* (2015)
IgG/TIV	Mouse/human	Influenza	Early production of sialylated IgG set the threshold for subsequent high‐affinity and protective antibody production.	Wang *et al* (2015b)
Chicken polyclonal serum/NDV	Chicken	Newcastle disease	Protection against viral challenge; some preparations reduced protection	Yosipovich *et al* (2015); Rautenschlein *et al* (2007); Pokric *et al* (1993)
bNAb/HIV gp120	Mouse (humanized)	HIV model	Longer period of aviremia after treatment; requires Fc portion for the effect	Halper‐Stromberg *et al* (2014)
Engineered bNAb/gp120	Mouse (humanized)	HIV model	Better protection against HIV entry; bNAb with Fc engineered to bind activating FcγR (humanized in mouse) are more effective	Bournazos *et al* (2014)
Specific mIgG2a/FT	Mouse	*Francisella tularensis*	Protection from subsequent challenges	Pham *et al* (2014)
Humanized mAb/RSV	Cotton rat	RSV	G0 glycosylation is linked to better protection against RSV challenge	Hiatt *et al* (2014)
Pre‐made HBsAg IC	Human	HBV	Induction of specific IgG1 and IgG3; reduced serum HBV DNA; reduced serum HBeAg; presence of anti‐HBeAg; increased HBeAg seroconversion	Xu *et al* (2013); Yao *et al* (2007); Xu *et al* (2005)
HBsAg fused to Fc of mIgG2a	Mouse	HBV model	Higher specific CD8 activation; elevated CD4 response	Hong *et al* (2011)
Specific mIgG2a and mIgG1/FrCasE virus	Mouse	Model of HIV maternal transmission	Reduced adolescent mortality from the virus; ADCC and CTL activations are involved	Michaud *et al* (2010)
Human IgG mAb/CD4 binding site of gp120	Mouse	HIV model	Higher anti‐gp120 titer; induction of neutralizing antibody	Hioe *et al* (2009); Visciano *et al* (2008)
Engineered specific hIgG1/*P. falciparum*	Mouse (humanized)	*Plasmodium berghei*	Protection from lethality; FcR‐binding/FcγRI are critical	McIntosh *et al* (2007)
Chicken polyclonal serum/IBDV	Chicken	Chicken Bursal Disease	Protection against viral challenge; immunization	Ivan *et al* (2005); Giambrone *et al* (2001); Jeurissen *et al* (1998); Haddad *et al* (1997)
IgG/HBsAg	Mouse	HBV model	HBsAb seroconversion in HBV transgenic mice	Zheng *et al* (2001)
Non‐neutralizing IgG fraction/SIV gp120	Rhesus monkey	SIV	No protection, FcγRIIB signaling, anti‐inflammatory gene expression	Polyanskaya *et al* (2001)
Matrixed rat anti‐DHBV/DHBV	Duck	DHBV protection	Reduced viral DNA and DHBsAg in serum	Wen *et al* (1994)
IgG/anti‐gp120 v3 loop	Human	HIV	Positive anti‐HIV proliferation of CD4 T cells	Berzofsky *et al* (1988)
Human polyclonal IgG/HBV HBsAg	Human	*In vitro* antibody production	Enhanced CD4 T‐cell activation; enhanced antibody production; requires Fc	Celis *et al* (1987); Celis and Chang (1984); Celis *et al* (1984)
Rabbit IgG/human or bovine albumin	Mouse	Vaccine efficacy	Enhanced model antigen destruction *in vivo*	Terres and Wolins (1959a, 1961)

**Pending issues**

**Precise mapping of Fc/FcγR subtype affinities**

Although the work has started, a complete map of subtype Fc/FcγR interaction affinities is not yet available. This map will be essential for precision targeting in IC/FcγR engagement in immune therapy and vaccination.

**How antigen presentation is regulated**

As ligation of subtypes of FcγRs results in native protein retention or MHC class I/II‐restricted antigen presentation, it is critical to systematically, using subtype Fc/FcγR pairing, determine the routing of antigen processing. This will provide a guideline for optimal induction of desired immune responses.

## Conflict of interest

The authors declare that they have no conflict of interest.

## References

[emmm201606593-bib-0001] Albert H , Collin M , Dudziak D , Ravetch JV , Nimmerjahn F (2008) *In vivo* enzymatic modulation of IgG glycosylation inhibits autoimmune disease in an IgG subclass‐dependent manner. Proc Natl Acad Sci USA 105: 15005–15009 18815375 10.1073/pnas.0808248105 PMC2567483

[emmm201606593-bib-0002] Amigorena S , Bonnerot C (1999) Fc receptors for IgG and antigen presentation on MHC class I and class II molecules. Semin Immunol 11: 385–390 10625592 10.1006/smim.1999.0196

[emmm201606593-bib-0003] Amigorena S , Savina A (2010) Intracellular mechanisms of antigen cross presentation in dendritic cells. Curr Opin Immunol 22: 109–117 20171863 10.1016/j.coi.2010.01.022

[emmm201606593-bib-0004] Astry CL , Jakab GJ (1984) Influenza virus‐induced immune complexes suppress alveolar macrophage phagocytosis. J Virol 50: 287–292 6708169 10.1128/jvi.50.2.287-292.1984 PMC255619

[emmm201606593-bib-0005] Bergtold A , Desai DD , Gavhane A , Clynes R (2005) Cell surface recycling of internalized antigen permits dendritic cell priming of B cells. Immunity 23: 503–514 16286018 10.1016/j.immuni.2005.09.013

[emmm201606593-bib-0006] Berken A , Benacerraf B (1966) Properties of antibodies cytophilic for macrophages. J Exp Med 123: 119–144 4159249 10.1084/jem.123.1.119 PMC2138129

[emmm201606593-bib-0007] Berlyn KA , Schultes B , Leveugle B , Noujaim AA , Alexander RB , Mann DL (2001) Generation of CD4(+) and CD8(+) T lymphocyte responses by dendritic cells armed with PSA/anti‐PSA (antigen/antibody) complexes. Clin Immunol 101: 276–283 11726219 10.1006/clim.2001.5115

[emmm201606593-bib-0008] Berzofsky JA , Bensussan A , Cease KB , Bourge JF , Cheynier R , Lurhuma Z , Salaun JJ , Gallo RC , Shearer GM , Zagury D (1988) Antigenic peptides recognized by T lymphocytes from AIDS viral envelope‐immune humans. Nature 334: 706–708 2457809 10.1038/334706a0

[emmm201606593-bib-0009] Bohm S , Kao D , Nimmerjahn F (2014) Sweet and sour: the role of glycosylation for the anti‐inflammatory activity of immunoglobulin G. Curr Top Microbiol Immunol 382: 393–417 25116110 10.1007/978-3-319-07911-0_18

[emmm201606593-bib-0010] Bonifaz LC , Bonnyay DP , Charalambous A , Darguste DI , Fujii S , Soares H , Brimnes MK , Moltedo B , Moran TM , Steinman RM (2004) *In vivo* targeting of antigens to maturing dendritic cells via the DEC‐205 receptor improves T cell vaccination. J Exp Med 199: 815–824 15024047 10.1084/jem.20032220 PMC2212731

[emmm201606593-bib-0011] Bonnerot C , Briken V , Brachet V , Lankar D , Cassard S , Jabri B , Amigorena S (1998) syk protein tyrosine kinase regulates Fc receptor gamma‐chain‐mediated transport to lysosomes. EMBO J 17: 4606–4616 9707420 10.1093/emboj/17.16.4606 PMC1170790

[emmm201606593-bib-0012] Boross P , van Montfoort N , Stapels DA , van der Poel CE , Bertens C , Meeldijk J , Jansen JH , Verbeek JS , Ossendorp F , Wubbolts R *et al* (2014) FcRgamma‐chain ITAM signaling is critically required for cross‐presentation of soluble antibody‐antigen complexes by dendritic cells. J Immunol 193: 5506–5514 25355925 10.4049/jimmunol.1302012

[emmm201606593-bib-0013] Bournazos S , Klein F , Pietzsch J , Seaman MS , Nussenzweig MC , Ravetch JV (2014) Broadly neutralizing anti‐HIV‐1 antibodies require Fc effector functions for *in vivo* activity. Cell 158: 1243–1253 25215485 10.1016/j.cell.2014.08.023 PMC4167398

[emmm201606593-bib-0014] Boyden SV , Sorkin E (1960) The adsorption of antigen by spleen cells previously treated with antiserum *in vitro* . Immunology 3: 272–283 13803569 PMC1424001

[emmm201606593-bib-0015] Buchbinder SP , Mehrotra DV , Duerr A , Fitzgerald DW , Mogg R , Li D , Gilbert PB , Lama JR , Marmor M , Del Rio C *et al*, (2008) Efficacy assessment of a cell‐mediated immunity HIV‐1 vaccine (the Step Study): a double‐blind, randomised, placebo‐controlled, test‐of‐concept trial. Lancet 372: 1881–1893 19012954 10.1016/S0140-6736(08)61591-3 PMC2721012

[emmm201606593-bib-0016] Burgdorf S , Kautz A , Bohnert V , Knolle PA , Kurts C (2007) Distinct pathways of antigen uptake and intracellular routing in CD4 and CD8 T cell activation. Science 316: 612–616 17463291 10.1126/science.1137971

[emmm201606593-bib-0017] Celis E , Abraham KG , Miller RW (1987) Modulation of the immunological response to hepatitis B virus by antibodies. Hepatology 7: 563–568 3494654 10.1002/hep.1840070324

[emmm201606593-bib-0018] Celis E , Chang TW (1984) Antibodies to hepatitis B surface antigen potentiate the response of human T lymphocyte clones to the same antigen. Science 224: 297–299 6231724 10.1126/science.6231724

[emmm201606593-bib-0019] Celis E , Zurawski VR Jr , Chang TW (1984) Regulation of T‐cell function by antibodies: enhancement of the response of human T‐cell clones to hepatitis B surface antigen by antigen‐specific monoclonal antibodies. Proc Natl Acad Sci USA 81: 6846–6850 6436821 10.1073/pnas.81.21.6846 PMC392029

[emmm201606593-bib-0020] Charalambous A , Oks M , Nchinda G , Yamazaki S , Steinman RM (2006) Dendritic cell targeting of survivin protein in a xenogeneic form elicits strong CD4+ T cell immunity to mouse survivin. J Immunol 177: 8410–8421 17142738 10.4049/jimmunol.177.12.8410

[emmm201606593-bib-0021] Chatterjee B , Smed‐Sorensen A , Cohn L , Chalouni C , Vandlen R , Lee BC , Widger J , Keler T , Delamarre L , Mellman I (2012) Internalization and endosomal degradation of receptor‐bound antigens regulate the efficiency of cross presentation by human dendritic cells. Blood 120: 2011–2020 22791285 10.1182/blood-2012-01-402370

[emmm201606593-bib-0022] Clatworthy MR , Aronin CE , Mathews RJ , Morgan NY , Smith KG , Germain RN (2014) Immune complexes stimulate CCR7‐dependent dendritic cell migration to lymph nodes. Nat Med 20: 1458–1463 25384086 10.1038/nm.3709 PMC4283039

[emmm201606593-bib-0023] Cohn L , Chatterjee B , Esselborn F , Smed‐Sorensen A , Nakamura N , Chalouni C , Lee BC , Vandlen R , Keler T , Lauer P *et al* (2013) Antigen delivery to early endosomes eliminates the superiority of human blood BDCA3+ dendritic cells at cross presentation. J Exp Med 210: 1049–1063 23569326 10.1084/jem.20121251 PMC3646496

[emmm201606593-bib-0024] Copeman SM , O'brien RA , Eagleton AJ , Glenny AT (1922) Experiences with the Schick Test and Active Immunization against Diphtheria. Br J Exp Pathol 3: 42 10.1177/003591572201501405 PMC2102196 19982498

[emmm201606593-bib-0025] Corti D , Misasi J , Mulangu S , Stanley DA , Kanekiyo M , Wollen S , Ploquin A , Doria‐Rose NA , Staupe RP , Bailey M *et al* (2016) Protective monotherapy against lethal Ebola virus infection by a potently neutralizing antibody. Science 351: 1339–1342 26917593 10.1126/science.aad5224

[emmm201606593-bib-0026] Devey ME , Steward MW (1980) The induction of chronic antigen‐antibody complex disease in selectively bred mice producing either high or low affinity antibody to protein antigens. Immunology 41: 303–311 6449473 PMC1458182

[emmm201606593-bib-0027] Dhodapkar KM , Krasovsky J , Williamson B , Dhodapkar MV (2002) Antitumor monoclonal antibodies enhance cross‐presentation of cellular antigens and the generation of myeloma‐specific killer T cells by dendritic cells. J Exp Med 195: 125–133 11781371 10.1084/jem.20011097 PMC2196013

[emmm201606593-bib-0028] DiLillo DJ , Ravetch JV (2015) Differential Fc‐Receptor Engagement Drives an Anti‐tumor Vaccinal Effect. Cell 161: 1035–1045 25976835 10.1016/j.cell.2015.04.016 PMC4441863

[emmm201606593-bib-0029] Ekiert DC , Friesen RH , Bhabha G , Kwaks T , Jongeneelen M , Yu W , Ophorst C , Cox F , Korse HJ , Brandenburg B *et al* (2011) A highly conserved neutralizing epitope on group 2 influenza A viruses. Science 333: 843–850 21737702 10.1126/science.1204839 PMC3210727

[emmm201606593-bib-0030] Giambrone JJ , Dormitorio T , Brown T (2001) Safety and efficacy of in ovo administration of infectious bursal disease viral vaccines. Avian Dis 45: 144–148 11332475

[emmm201606593-bib-0031] Goncalvez AP , Engle RE , St Claire M , Purcell RH , Lai CJ (2007) Monoclonal antibody‐mediated enhancement of dengue virus infection *in vitro* and *in vivo* and strategies for prevention. Proc Natl Acad Sci USA 104: 9422–9427 17517625 10.1073/pnas.0703498104 PMC1868655

[emmm201606593-bib-0032] Guilliams M , Bruhns P , Saeys Y , Hammad H , Lambrecht BN (2014) The function of Fcgamma receptors in dendritic cells and macrophages. Nat Rev Immunol 14: 94–108 24445665 10.1038/nri3582

[emmm201606593-bib-0033] den Haan JM , Bevan MJ (2002) Constitutive versus activation‐dependent cross‐presentation of immune complexes by CD8(+) and CD8(‐) dendritic cells *in vivo* . J Exp Med 196: 817–827 12235214 10.1084/jem.20020295 PMC2194052

[emmm201606593-bib-0034] Haddad EE , Whitfill CE , Avakian AP , Ricks CA , Andrews PD , Thoma JA , Wakenell PS (1997) Efficacy of a novel infectious bursal disease virus immune complex vaccine in broiler chickens. Avian Dis 41: 882–889 9454922

[emmm201606593-bib-0035] Halper‐Stromberg A , Lu CL , Klein F , Horwitz JA , Bournazos S , Nogueira L , Eisenreich TR , Liu C , Gazumyan A , Schaefer U *et al* (2014) Broadly neutralizing antibodies and viral inducers decrease rebound from HIV‐1 latent reservoirs in humanized mice. Cell 158: 989–999 25131989 10.1016/j.cell.2014.07.043 PMC4163911

[emmm201606593-bib-0036] Harbers SO , Crocker A , Catalano G , D'Agati V , Jung S , Desai DD , Clynes R (2007) Antibody‐enhanced cross‐presentation of self antigen breaks T cell tolerance. J Clin Invest 117: 1361–1369 17446931 10.1172/JCI29470 PMC1849985

[emmm201606593-bib-0037] Hari A , Ganguly A , Mu L , Davis SP , Stenner MD , Lam R , Munro F , Namet I , Alghamdi E , Furstenhaupt T *et al* (2015) Redirecting soluble antigen for MHC class I cross‐presentation during phagocytosis. Eur J Immunol 45: 383–395 25378230 10.1002/eji.201445156 PMC4324331

[emmm201606593-bib-0038] Heesters BA , Chatterjee P , Kim YA , Gonzalez SF , Kuligowski MP , Kirchhausen T , Carroll MC (2013) Endocytosis and recycling of immune complexes by follicular dendritic cells enhances B cell antigen binding and activation. Immunity 38: 1164–1175 23770227 10.1016/j.immuni.2013.02.023 PMC3773956

[emmm201606593-bib-0039] Hiatt A , Bohorova N , Bohorov O , Goodman C , Kim D , Pauly MH , Velasco J , Whaley KJ , Piedra PA , Gilbert BE *et al* (2014) Glycan variants of a respiratory syncytial virus antibody with enhanced effector function and *in vivo* efficacy. Proc Natl Acad Sci USA 111: 5992–5997 24711420 10.1073/pnas.1402458111 PMC4000855

[emmm201606593-bib-0040] Hioe CE , Visciano ML , Kumar R , Liu J , Mack EA , Simon RE , Levy DN , Tuen M (2009) The use of immune complex vaccines to enhance antibody responses against neutralizing epitopes on HIV‐1 envelope gp120. Vaccine 28: 352–360 19879224 10.1016/j.vaccine.2009.10.040 PMC2789659

[emmm201606593-bib-0041] Hong Y , Peng Y , Mi M , Xiao H , Munn DH , Wang GQ , He Y (2011) Lentivector expressing HBsAg and immunoglobulin Fc fusion antigen induces potent immune responses and results in seroconversion in HBsAg transgenic mice. Vaccine 29: 3909–3916 21421003 10.1016/j.vaccine.2011.03.025 PMC3090480

[emmm201606593-bib-0042] Horwitz JA , Halper‐Stromberg A , Mouquet H , Gitlin AD , Tretiakova A , Eisenreich TR , Malbec M , Gravemann S , Billerbeck E , Dorner M *et al* (2013) HIV‐1 suppression and durable control by combining single broadly neutralizing antibodies and antiretroviral drugs in humanized mice. Proc Natl Acad Sci USA 110: 16538–16543 24043801 10.1073/pnas.1315295110 PMC3799352

[emmm201606593-bib-0043] Iankov ID , Pandey M , Harvey M , Griesmann GE , Federspiel MJ , Russell SJ (2006) Immunoglobulin g antibody‐mediated enhancement of measles virus infection can bypass the protective antiviral immune response. J Virol 80: 8530–8540 16912303 10.1128/JVI.00593-06 PMC1563851

[emmm201606593-bib-0044] Ivan J , Velhner M , Ursu K , German P , Mato T , Dren CN , Meszaros J (2005) Delayed vaccine virus replication in chickens vaccinated subcutaneously with an immune complex infectious bursal disease vaccine: quantification of vaccine virus by real‐time polymerase chain reaction. Can J Vet Res 69: 135–142 15971678 PMC1142181

[emmm201606593-bib-0045] Jeurissen SH , Janse EM , Lehrbach PR , Haddad EE , Avakian A , Whitfill CE (1998) The working mechanism of an immune complex vaccine that protects chickens against infectious bursal disease. Immunology 95: 494–500 9824516 10.1046/j.1365-2567.1998.00617.x PMC1364419

[emmm201606593-bib-0046] Joffre OP , Segura E , Savina A , Amigorena S (2012) Cross‐presentation by dendritic cells. Nat Rev Immunol 12: 557–569 22790179 10.1038/nri3254

[emmm201606593-bib-0047] Kalergis AM , Ravetch JV (2002) Inducing tumor immunity through the selective engagement of activating Fcgamma receptors on dendritic cells. J Exp Med 195: 1653–1659 12070293 10.1084/jem.20020338 PMC2193555

[emmm201606593-bib-0048] Karsten CM , Pandey MK , Figge J , Kilchenstein R , Taylor PR , Rosas M , McDonald JU , Orr SJ , Berger M , Petzold D *et al* (2012) Anti‐inflammatory activity of IgG1 mediated by Fc galactosylation and association of FcgammaRIIB and dectin‐1. Nat Med 18: 1401–1406 22922409 10.1038/nm.2862 PMC3492054

[emmm201606593-bib-0049] Kim PS , Armstrong TD , Song H , Wolpoe ME , Weiss V , Manning EA , Huang LQ , Murata S , Sgouros G , Emens LA *et al* (2008) Antibody association with HER‐2/neu‐targeted vaccine enhances CD8 T cell responses in mice through Fc‐mediated activation of DCs. J Clin Invest 118: 1700–1711 18398507 10.1172/JCI34333 PMC2289797

[emmm201606593-bib-0050] Kosco MH , Burton GF , Kapasi ZF , Szakal AK , Tew JG (1989) Antibody‐forming cell induction during an early phase of germinal centre development and its delay with ageing. Immunology 68: 312–318 2592007 PMC1385441

[emmm201606593-bib-0051] Kotsias F , Hoffmann E , Amigorena S , Savina A (2013) Reactive oxygen species production in the phagosome: impact on antigen presentation in dendritic cells. Antioxid Redox Signal 18: 714–729 22827577 10.1089/ars.2012.4557

[emmm201606593-bib-0052] Koyama A , Niwa Y , Shigematsu H , Taniguchi M , Tada T (1978) Studies on passive serum sickness. II. Factors determining the localization of antigen‐antibody complexes in the murine renal glomerulus. Lab Invest 38: 253–262 633850

[emmm201606593-bib-0053] Kwong PD , Doyle ML , Casper DJ , Cicala C , Leavitt SA , Majeed S , Steenbeke TD , Venturi M , Chaiken I , Fung M *et al* (2002) HIV‐1 evades antibody‐mediated neutralization through conformational masking of receptor‐binding sites. Nature 420: 678–682 12478295 10.1038/nature01188

[emmm201606593-bib-0054] Le Roux D , Lankar D , Yuseff MI , Vascotto F , Yokozeki T , Faure‐Andre G , Mougneau E , Glaichenhaus N , Manoury B , Bonnerot C *et al* (2007) Syk‐dependent actin dynamics regulate endocytic trafficking and processing of antigens internalized through the B‐cell receptor. Mol Biol Cell 18: 3451–3462 17596518 10.1091/mbc.E06-12-1114 PMC1951757

[emmm201606593-bib-0055] Leon B , Ballesteros‐Tato A , Randall TD , Lund FE (2014) Prolonged antigen presentation by immune complex‐binding dendritic cells programs the proliferative capacity of memory CD8 T cells. J Exp Med 211: 1637–1655 25002751 10.1084/jem.20131692 PMC4113940

[emmm201606593-bib-0056] Liao HX , Alam SM , Mascola JR , Robinson J , Ma B , Montefiori DC , Rhein M , Sutherland LL , Scearce R , Haynes BF (2004) Immunogenicity of constrained monoclonal antibody A32‐human immunodeficiency virus (HIV) Env gp120 complexes compared to that of recombinant HIV type 1 gp120 envelope glycoproteins. J Virol 78: 5270–5278 15113908 10.1128/JVI.78.10.5270-5278.2004 PMC400342

[emmm201606593-bib-0057] Liu H , Geng S , Wang B , Wu B , Xie X , Wang S , Zhong Y , Wang X , Qu D , Wen Y *et al* (2016) Immuno‐potentiating pathway of HBsAg‐HBIG immunogenic complex visualized. Hum Vaccin Immunother 12: 77–84 26618396 10.1080/21645515.2015.1072660 PMC4962741

[emmm201606593-bib-0058] Lizee G , Basha G , Tiong J , Julien JP , Tian M , Biron KE , Jefferies WA (2003) Control of dendritic cell cross‐presentation by the major histocompatibility complex class I cytoplasmic domain. Nat Immunol 4: 1065–1073 14566337 10.1038/ni989

[emmm201606593-bib-0059] Lu L , Xia S , Ying T , Jiang S (2015) Urgent development of effective therapeutic and prophylactic agents to control the emerging threat of Middle East respiratory syndrome (MERS). Emerg Microbes Infect 4: e37 26954884 10.1038/emi.2015.37 PMC4773045

[emmm201606593-bib-0060] Matsubara S , Koya T , Takeda K , Joetham A , Miyahara N , Pine P , Masuda ES , Swasey CH , Gelfand EW (2006) Syk activation in dendritic cells is essential for airway hyperresponsiveness and inflammation. Am J Respir Cell Mol Biol 34: 426–433 16339999 10.1165/rcmb.2005-0298OC PMC2644204

[emmm201606593-bib-0061] McClintock SD , Barron AG , Olle EW , Deogracias MP , Warner RL , Opp M , Johnson KJ (2005) Role of interleukin‐6 in immune complex induced models of vascular injury. Inflammation 29: 154–162 17089190 10.1007/s10753-006-9011-1

[emmm201606593-bib-0062] McEwan WA , Tam JC , Watkinson RE , Bidgood SR , Mallery DL , James LC (2013) Intracellular antibody‐bound pathogens stimulate immune signaling via the Fc receptor TRIM21. Nat Immunol 14: 327–336 23455675 10.1038/ni.2548 PMC3672961

[emmm201606593-bib-0063] McIntosh RS , Shi J , Jennings RM , Chappel JC , de Koning‐Ward TF , Smith T , Green J , van Egmond M , Leusen JH , Lazarou M *et al* (2007) The importance of human FcgammaRI in mediating protection to malaria. PLoS Pathog 3: e72 17511516 10.1371/journal.ppat.0030072 PMC1868954

[emmm201606593-bib-0064] Meng ZF , Wang HJ , Yao X , Wang XY , Wen YM , Dai JX , Xie YH , Xu JQ (2012) Immunization with HBsAg‐Fc fusion protein induces a predominant production of Th1 cytokines and reduces HBsAg level in transgenic mice. Chin Med J (Engl) 125: 3266–3272 22964321

[emmm201606593-bib-0065] Michaud HA , Gomard T , Gros L , Thiolon K , Nasser R , Jacquet C , Hernandez J , Piechaczyk M , Pelegrin M (2010) A crucial role for infected‐cell/antibody immune complexes in the enhancement of endogenous antiviral immunity by short passive immunotherapy. PLoS Pathog 6: e1000948 20548955 10.1371/journal.ppat.1000948 PMC2883599

[emmm201606593-bib-0066] Moalli F , Doni A , Deban L , Zelante T , Zagarella S , Bottazzi B , Romani L , Mantovani A , Garlanda C (2010) Role of complement and Fcγ receptors in the protective activity of the long pentraxin PTX3 against Aspergillus fumigatus. Blood 116: 5170–5180 20829368 10.1182/blood-2009-12-258376

[emmm201606593-bib-0067] Mocsai A , Ruland J , Tybulewicz VL (2010) The SYK tyrosine kinase: a crucial player in diverse biological functions. Nat Rev Immunol 10: 387–402 20467426 10.1038/nri2765 PMC4782221

[emmm201606593-bib-0068] Neubert K , Lehmann CH , Heger L , Baranska A , Staedtler AM , Buchholz VR , Yamazaki S , Heidkamp GF , Eissing N , Zebroski H *et al* (2014) Antigen delivery to CD11c+CD8‐ dendritic cells induces protective immune responses against experimental melanoma in mice *in vivo* . J Immunol 192: 5830–5838 24829411 10.4049/jimmunol.1300975

[emmm201606593-bib-0069] Nimmerjahn F , Ravetch JV (2005) Divergent immunoglobulin g subclass activity through selective Fc receptor binding. Science 310: 1510–1512 16322460 10.1126/science.1118948

[emmm201606593-bib-0070] Nimmerjahn F , Ravetch JV (2007) Fc‐receptors as regulators of immunity. Adv Immunol 96: 179–204 17981207 10.1016/S0065-2776(07)96005-8

[emmm201606593-bib-0071] Nimmerjahn F , Ravetch JV (2008) Fcgamma receptors as regulators of immune responses. Nat Rev Immunol 8: 34–47 18064051 10.1038/nri2206

[emmm201606593-bib-0072] Nimmerjahn F , Ravetch JV (2012) Translating basic mechanisms of IgG effector activity into next generation cancer therapies. Cancer Immun 12: 13 22896758 PMC3380343

[emmm201606593-bib-0073] Noguchi T , Kato T , Wang L , Maeda Y , Ikeda H , Sato E , Knuth A , Gnjatic S , Ritter G , Sakaguchi S *et al* (2012) Intracellular tumor‐associated antigens represent effective targets for passive immunotherapy. Cancer Res 72: 1672–1682 22318866 10.1158/0008-5472.CAN-11-3072

[emmm201606593-bib-0074] Nydegger UE (2007) Immune complex pathophysiology. Ann NY Acad Sci 1109: 66–83 17785292 10.1196/annals.1398.009

[emmm201606593-bib-0075] Oefner CM , Winkler A , Hess C , Lorenz AK , Holecska V , Huxdorf M , Schommartz T , Petzold D , Bitterling J , Schoen AL *et al* (2012) Tolerance induction with T cell‐dependent protein antigens induces regulatory sialylated IgGs. J Allergy Clin Immunol 129(1647–1655): e1613 10.1016/j.jaci.2012.02.037 22502800

[emmm201606593-bib-0076] Okuno Y , Isegawa Y , Sasao F , Ueda S (1993) A common neutralizing epitope conserved between the hemagglutinins of influenza A virus H1 and H2 strains. J Virol 67: 2552–2558 7682624 10.1128/jvi.67.5.2552-2558.1993 PMC237575

[emmm201606593-bib-0077] Okuno Y , Matsumoto K , Isegawa Y , Ueda S (1994) Protection against the mouse‐adapted A/FM/1/47 strain of influenza A virus in mice by a monoclonal antibody with cross‐neutralizing activity among H1 and H2 strains. J Virol 68: 517–520 8254764 10.1128/jvi.68.1.517-520.1994 PMC236314

[emmm201606593-bib-0078] Olitzki L (1935) The antigenic properties of bacteria combined with antibodies. J Immunol 29: 453–465

[emmm201606593-bib-0079] Pan R , Chen Y , Vaine M , Hu G , Wang S , Lu S , Kong XP (2015) Structural analysis of a novel rabbit monoclonal antibody R53 targeting an epitope in HIV‐1 gp120 C4 region critical for receptor and co‐receptor binding. Emerg Microbes Infect 4: e44 26251831 10.1038/emi.2015.44 PMC4522616

[emmm201606593-bib-0080] Park‐Min KH , Serbina NV , Yang W , Ma X , Krystal G , Neel BG , Nutt SL , Hu X , Ivashkiv LB (2007) FcgammaRIII‐dependent inhibition of interferon‐gamma responses mediates suppressive effects of intravenous immune globulin. Immunity 26: 67–78 17239631 10.1016/j.immuni.2006.11.010

[emmm201606593-bib-0081] Perreau M , Pantaleo G , Kremer EJ (2008) Activation of a dendritic cell‐T cell axis by Ad5 immune complexes creates an improved environment for replication of HIV in T cells. J Exp Med 205: 2717–2725 18981239 10.1084/jem.20081786 PMC2585831

[emmm201606593-bib-0082] Pham GH , Iglesias BV , Gosselin EJ (2014) Fc receptor‐targeting of immunogen as a strategy for enhanced antigen loading, vaccination, and protection using intranasally administered antigen‐pulsed dendritic cells. Vaccine 32: 5212–5220 25068496 10.1016/j.vaccine.2014.07.050 PMC4144271

[emmm201606593-bib-0083] Phan TG , Grigorova I , Okada T , Cyster JG (2007) Subcapsular encounter and complement‐dependent transport of immune complexes by lymph node B cells. Nat Immunol 8: 992–1000 17660822 10.1038/ni1494

[emmm201606593-bib-0084] Phoolcharoen W , Bhoo SH , Lai H , Ma J , Arntzen CJ , Chen Q , Mason HS (2011) Expression of an immunogenic Ebola immune complex in Nicotiana benthamiana. Plant Biotechnol J 9: 807–816 21281425 10.1111/j.1467-7652.2011.00593.x PMC4022790

[emmm201606593-bib-0085] Pincetic A , Bournazos S , DiLillo DJ , Maamary J , Wang TT , Dahan R , Fiebiger BM , Ravetch JV (2014) Type I and type II Fc receptors regulate innate and adaptive immunity. Nat Immunol 15: 707–716 25045879 10.1038/ni.2939 PMC7430760

[emmm201606593-bib-0086] Platzer B , Stout M , Fiebiger E (2014) Antigen cross‐presentation of immune complexes. Front Immunol 5: 140 24744762 10.3389/fimmu.2014.00140 PMC3978348

[emmm201606593-bib-0087] Pleass RJ (2009) Fc‐receptors and immunity to malaria: from models to vaccines. Parasite Immunol 31: 529–538 19691552 10.1111/j.1365-3024.2009.01101.x PMC3115686

[emmm201606593-bib-0088] Pokric B , Sladic D , Juros S , Cajavec S (1993) Application of the immune complex for immune protection against viral disease. Vaccine 11: 655–659 8322489 10.1016/0264-410x(93)90312-l

[emmm201606593-bib-0089] Polyanskaya N , Bergmeier LA , Sharpe SA , Cook N , Leech S , Hall G , Dennis M , ten Haaft P , Heeney J , Manca F *et al* (2001) Mucosal exposure to subinfectious doses of SIV primes gut‐associated antibody‐secreting cells and T cells: lack of enhancement by nonneutralizing antibody. Virology 279: 527–538 11162808 10.1006/viro.2000.0704

[emmm201606593-bib-0090] Qin D , Wu J , Vora KA , Ravetch JV , Szakal AK , Manser T , Tew JG (2000) Fc gamma receptor IIB on follicular dendritic cells regulates the B cell recall response. J Immunol 164: 6268–6275 10843680 10.4049/jimmunol.164.12.6268

[emmm201606593-bib-0091] Rafiq K , Bergtold A , Clynes R (2002) Immune complex‐mediated antigen presentation induces tumor immunity. J Clin Invest 110: 71–79 12093890 10.1172/JCI15640 PMC151032

[emmm201606593-bib-0092] Rautenschlein S , Kraemer C , Montiel E , Vanmarcke J , Haase C (2007) Bilateral effects of vaccination against infectious bursal disease and Newcastle disease in specific‐pathogen‐free layers and commercial broiler chickens. Avian Dis 51: 14–20 17461261 10.1637/0005-2086(2007)051[0014:BEOVAI]2.0.CO;2

[emmm201606593-bib-0093] Ravetch JV , Kinet JP (1991) Fc receptors. Annu Rev Immunol 9: 457–492 1910686 10.1146/annurev.iy.09.040191.002325

[emmm201606593-bib-0094] Ravetch J (2010) *In vivo* veritas: the surprising roles of Fc receptors in immunity. Nat Immunol 11: 183–185 20157296 10.1038/ni0310-183

[emmm201606593-bib-0095] Regnault A , Lankar D , Lacabanne V , Rodriguez A , Thery C , Rescigno M , Saito T , Verbeek S , Bonnerot C , Ricciardi‐Castagnoli P *et al* (1999) Fcgamma receptor‐mediated induction of dendritic cell maturation and major histocompatibility complex class I‐restricted antigen presentation after immune complex internalization. J Exp Med 189: 371–380 9892619 10.1084/jem.189.2.371 PMC2192989

[emmm201606593-bib-0096] Sancho D , Mourao‐Sa D , Joffre OP , Schulz O , Rogers NC , Pennington DJ , Carlyle JR , Reis e Sousa C (2008) Tumor therapy in mice via antigen targeting to a novel, DC‐restricted C‐type lectin. J Clin Invest 118: 2098–2110 18497879 10.1172/JCI34584 PMC2391066

[emmm201606593-bib-0097] Schuurhuis DH , Ioan‐Facsinay A , Nagelkerken B , van Schip JJ , Sedlik C , Melief CJ , Verbeek JS , Ossendorp F (2002) Antigen‐antibody immune complexes empower dendritic cells to efficiently prime specific CD8+ CTL responses *in vivo* . J Immunol 168: 2240–2246 11859111 10.4049/jimmunol.168.5.2240

[emmm201606593-bib-0098] Schwab I , Nimmerjahn F (2013) Intravenous immunoglobulin therapy: how does IgG modulate the immune system? Nat Rev Immunol 13: 176–189 23411799 10.1038/nri3401

[emmm201606593-bib-0099] Scott AM , Wolchok JD , Old LJ (2012) Antibody therapy of cancer. Nat Rev Cancer 12: 278–287 22437872 10.1038/nrc3236

[emmm201606593-bib-0100] Sedlik C , Orbach D , Veron P , Schweighoffer E , Colucci F , Gamberale R , Ioan‐Facsinay A , Verbeek S , Ricciardi‐Castagnoli P , Bonnerot C *et al* (2003) A critical role for Syk protein tyrosine kinase in Fc receptor‐mediated antigen presentation and induction of dendritic cell maturation. J Immunol 170: 846–852 12517949 10.4049/jimmunol.170.2.846

[emmm201606593-bib-0101] Shakushiro K , Yamasaki Y , Nishikawa M , Takakura Y (2004) Efficient scavenger receptor‐mediated uptake and cross‐presentation of negatively charged soluble antigens by dendritic cells. Immunology 112: 211–218 15147564 10.1111/j.1365-2567.2004.01871.x PMC1782477

[emmm201606593-bib-0102] Sondak VK , Smalley KS , Kudchadkar R , Grippon S , Kirkpatrick P (2011) Ipilimumab. Nat Rev Drug Discov 10: 411–412 21629286 10.1038/nrd3463

[emmm201606593-bib-0103] St Pierre CA , Leonard D , Corvera S , Kurt‐Jones EA , Finberg RW (2011) Antibodies to cell surface proteins redirect intracellular trafficking pathways. Exp Mol Pathol 91: 723–732 21819978 10.1016/j.yexmp.2011.05.011 PMC3315679

[emmm201606593-bib-0104] Suhrbier A , La Linn M (2003) Suppression of antiviral responses by antibody‐dependent enhancement of macrophage infection. Trends Immunol 24: 165–168 12697441 10.1016/s1471-4906(03)00065-6

[emmm201606593-bib-0105] Szakal AK , Kosco MH , Tew JG (1988a) FDC‐iccosome mediated antigen delivery to germinal center B cells, antigen processing and presentation to T cells. Adv Exp Med Biol 237: 197–202 3267045 10.1007/978-1-4684-5535-9_29

[emmm201606593-bib-0106] Szakal AK , Kosco MH , Tew JG (1988b) A novel *in vivo* follicular dendritic cell‐dependent iccosome‐mediated mechanism for delivery of antigen to antigen‐processing cells. J Immunol 140: 341–353 3257233

[emmm201606593-bib-0107] Terres G , Hughes WL , Wolins W (1960) Whole‐body measurement of radioactivity as a means of following *in vivo* the degradation of I‐131‐labeled proteins in mice. Am J Physiol 198: 1355–1360 13837510 10.1152/ajplegacy.1960.198.6.1355

[emmm201606593-bib-0108] Terres G , Wolins W (1959a) Enhanced sensitization in mice by simultaneous injection of antigen and specific rabbit antiserum. Proc Soc Exp Biol Med 102: 632–635 13837512 10.3181/00379727-102-25342

[emmm201606593-bib-0109] Terres G , Wolins W (1959b) Immune degradation in passively sensitized mice. I. Degradation of antigen as a function of the amount of antigen and antibody used. J Immunol 83: 9–16 13665008

[emmm201606593-bib-0110] Terres G , Wolins W (1961) Enhanced immunological sensitization of mice by the simultaneous injection of antigen and specific antiserum. I. Effect of varying the amount of antigen used relative to the antiserum. J Immunol 86: 361–368 13776027

[emmm201606593-bib-0111] Tirado SM , Yoon KJ (2003) Antibody‐dependent enhancement of virus infection and disease. Viral Immunol 16: 69–86 12725690 10.1089/088282403763635465

[emmm201606593-bib-0112] Vasovic LV , Dyall R , Clynes RA , Ravetch JV , Nikolic‐Zugic J (1997) Synergy between an antibody and CD8+ cells in eliminating an established tumor. Eur J Immunol 27: 374–382 9045907 10.1002/eji.1830270206

[emmm201606593-bib-0113] Visciano ML , Tuen M , Gorny MK , Hioe CE (2008) *In vivo* alteration of humoral responses to HIV‐1 envelope glycoprotein gp120 by antibodies to the CD4‐binding site of gp120. Virology 372: 409–420 18054978 10.1016/j.virol.2007.10.044 PMC2288784

[emmm201606593-bib-0114] Walker LM , Phogat SK , Chan‐Hui PY , Wagner D , Phung P , Goss JL , Wrin T , Simek MD , Fling S , Mitcham JL *et al* (2009) Broad and potent neutralizing antibodies from an African donor reveal a new HIV‐1 vaccine target. Science 326: 285–289 19729618 10.1126/science.1178746 PMC3335270

[emmm201606593-bib-0115] Wang B , Zaidi N , He LZ , Zhang L , Kuroiwa JM , Keler T , Steinman RM (2012) Targeting of the non‐mutated tumor antigen HER2/neu to mature dendritic cells induces an integrated immune response that protects against breast cancer in mice. Breast Cancer Res 14: R39 22397502 10.1186/bcr3135 PMC3446373

[emmm201606593-bib-0116] Wang JR , Guan WD , Yau LF , Gao WN , Zhan YQ , Liu L , Yang ZF , Jiang ZH (2015a) Glycomic signatures on serum IgGs for prediction of postvaccination response. Sci Rep 5: 7648 25612906 10.1038/srep07648 PMC4303884

[emmm201606593-bib-0117] Wang TT , Maamary J , Tan GS , Bournazos S , Davis CW , Krammer F , Schlesinger SJ , Palese P , Ahmed R , Ravetch JV (2015b) Anti‐HA Glycoforms Drive B Cell Affinity Selection and Determine Influenza Vaccine Efficacy. Cell 162: 160–169 26140596 10.1016/j.cell.2015.06.026 PMC4594835

[emmm201606593-bib-0118] Warren JS , Yabroff KR , Remick DG , Kunkel SL , Chensue SW , Kunkel RG , Johnson KJ , Ward PA (1989) Tumor necrosis factor participates in the pathogenesis of acute immune complex alveolitis in the rat. J Clin Invest 84: 1873–1882 2531759 10.1172/JCI114374 PMC304067

[emmm201606593-bib-0119] Wen YM , Xiong SD , Zhang W (1994) Solid matrix‐antibody‐antigen complex can clear viraemia and antigenaemia in persistent duck hepatitis B virus infection. J Gen Virol 75: 335–339 8113755 10.1099/0022-1317-75-2-335

[emmm201606593-bib-0120] Wen YM , Wu XH , Hu DC , Zhang QP , Guo SQ (1995) Hepatitis B vaccine and anti‐HBs complex as approach for vaccine therapy. Lancet 345: 1575–1576 7791465 10.1016/s0140-6736(95)91126-x

[emmm201606593-bib-0121] Wieland A , Shashidharamurthy R , Kamphorst AO , Han JH , Aubert RD , Choudhury BP , Stowell SR , Lee J , Punkosdy GA , Shlomchik MJ *et al* (2015) Antibody effector functions mediated by Fcgamma‐receptors are compromised during persistent viral infection. Immunity 42: 367–378 25680276 10.1016/j.immuni.2015.01.009 PMC4339104

[emmm201606593-bib-0122] Wolpoe ME , Lutz ER , Ercolini AM , Murata S , Ivie SE , Garrett ES , Emens LA , Jaffee EM , Reilly RT (2003) HER‐2/neu‐specific monoclonal antibodies collaborate with HER‐2/neu‐targeted granulocyte macrophage colony‐stimulating factor secreting whole cell vaccination to augment CD8+ T cell effector function and tumor‐free survival in Her‐2/neu‐transgenic mice. J Immunol 171: 2161–2169 12902523 10.4049/jimmunol.171.4.2161

[emmm201606593-bib-0123] Wu X , Yang ZY , Li Y , Hogerkorp CM , Schief WR , Seaman MS , Zhou T , Schmidt SD , Wu L , Xu L *et al* (2010) Rational design of envelope identifies broadly neutralizing human monoclonal antibodies to HIV‐1. Science 329: 856–861 20616233 10.1126/science.1187659 PMC2965066

[emmm201606593-bib-0124] Wu Y , Cho M , Shore D , Song M , Choi J , Jiang T , Deng YQ , Bourgeois M , Almli L , Yang H *et al* (2015) A potent broad‐spectrum protective human monoclonal antibody crosslinking two haemagglutinin monomers of influenza A virus. Nat Commun 6: 7708 26196962 10.1038/ncomms8708 PMC4518248

[emmm201606593-bib-0125] Wu Y , Sukumar S , El Shikh ME , Best AM , Szakal AK , Tew JG (2008) Immune complex‐bearing follicular dendritic cells deliver a late antigenic signal that promotes somatic hypermutation. J Immunol 180: 281–290 18097029 10.4049/jimmunol.180.1.281

[emmm201606593-bib-0126] Xu DZ , Huang KL , Zhao K , Xu LF , Shi N , Yuan ZH , Wen YM (2005) Vaccination with recombinant HBsAg‐HBIG complex in healthy adults. Vaccine 23: 2658–2664 15780449 10.1016/j.vaccine.2004.10.040

[emmm201606593-bib-0127] Xu DZ , Zhao K , Guo LM , Li LJ , Xie Q , Ren H , Zhang JM , Xu M , Wang HF , Huang WX *et al* (2008) A randomized controlled phase IIb trial of antigen‐antibody immunogenic complex therapeutic vaccine in chronic hepatitis B patients. PLoS One 3: e2565 18596958 10.1371/journal.pone.0002565 PMC2430617

[emmm201606593-bib-0128] Xu DZ , Wang XY , Shen XL , Gong GZ , Ren H , Guo LM , Sun AM , Xu M , Li LJ , Guo XH *et al,* (2013) Results of a phase III clinical trial with an HBsAg‐HBIG immunogenic complex therapeutic vaccine for chronic hepatitis B patients: experiences and findings. J Hepatol 59: 450–456 23669281 10.1016/j.jhep.2013.05.003

[emmm201606593-bib-0129] Yamada DH , Elsaesser H , Lux A , Timmerman JM , Morrison SL , de la Torre JC , Nimmerjahn F , Brooks DG (2015) Suppression of Fcgamma‐receptor‐mediated antibody effector function during persistent viral infection. Immunity 42: 379–390 25680277 10.1016/j.immuni.2015.01.005 PMC4334737

[emmm201606593-bib-0130] Yao X , Zheng B , Zhou J , Xu DZ , Zhao K , Sun SH , Yuan ZH , Wen YM (2007) Therapeutic effect of hepatitis B surface antigen‐antibody complex is associated with cytolytic and non‐cytolytic immune responses in hepatitis B patients. Vaccine 25: 1771–1779 17224217 10.1016/j.vaccine.2006.11.019

[emmm201606593-bib-0131] Yosipovich R , Aizenshtein E , Shadmon R , Krispel S , Shuster E , Pitcovski J (2015) Overcoming the susceptibility gap between maternal antibody disappearance and auto‐antibody production. Vaccine 33: 472–478 25444785 10.1016/j.vaccine.2014.10.043

[emmm201606593-bib-0132] Zheng B , Zhang Y , He H , Marinova E , Switzer K , Wansley D , Mbawuike I , Han S (2007) Rectification of age‐associated deficiency in cytotoxic T cell response to influenza A virus by immunization with immune complexes. J Immunol 179: 6153–6159 17947690 10.4049/jimmunol.179.9.6153

[emmm201606593-bib-0133] Zheng BJ , Ng MH , He LF , Yao X , Chan KW , Yuen KY , Wen YM (2001) Therapeutic efficacy of hepatitis B surface antigen‐antibodies‐recombinant DNA composite in HBsAg transgenic mice. Vaccine 19: 4219–4225 11457548 10.1016/s0264-410x(01)00158-x

